# Emerging risks from marine heat waves

**DOI:** 10.1038/s41467-018-03163-6

**Published:** 2018-02-13

**Authors:** Thomas L. Frölicher, Charlotte Laufkötter

**Affiliations:** 1 Climate and Environmental Physics, Physics Institute, University of Bern, Sidlerstrasse 5, Bern, 3012 Switzerland; 2 Oeschger Centre for Climate Change Research, University of Bern, Bern, Switzerland

## Abstract

Recent marine heat waves have caused devastating impacts on marine ecosystems. Substantial progress in understanding past and future changes in marine heat waves and their risks for marine ecosystems is needed to predict how marine systems, and the goods and services they provide, will evolve in the future.

Extreme climate and weather events shape the structure of terrestrial biological systems and affect the biogeochemical functions and services they provide for society in a fundamental manner^1^. There is overwhelming evidence that atmospheric heat waves over land are changing under global warming, increasing the risk of severe, pervasive and in some cases irreversible impacts on natural and socio-economic systems^2^. In contrast, we know little how extreme events in the ocean, especially those associated with warming will change under global warming, and how they will impact marine organisms. This knowledge gap is of particular concern as some of the recent observed marine heat waves (MHWs) demonstrated the high vulnerability of marine organisms and ecosystems services to such extreme climate events.

## Definition, observations, and key processes

**Fig. 1:**
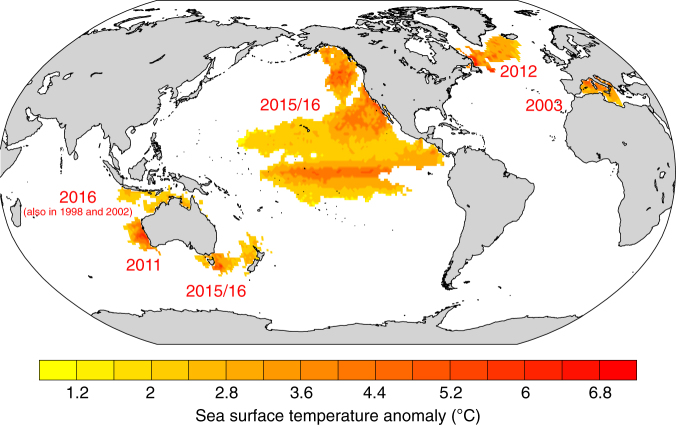
Summary of prominent recent marine heat waves that are documented and analyzed in the literature. The figure shows the maximum sea surface temperature anomaly in regions where temperature exceeds the 99th percentile using NOAA’s daily Optimum Interpolation sea surface temperature dataset^11^. The numbers indicate the year of the MHW occurrence. The 99th percentile is calculated over the 1982–2016 reference period. The map was created using the NCAR Command Language (https://www.ncl.ucar.edu)

A marine heat wave is usually defined as a coherent area of extreme warm sea surface temperature (SST) that persists for days to months^3^. MHWs have been observed in all major ocean basins over the recent decade, but only a few MHWs have been documented and analyzed extensively (Fig. 1). One of the first MHW that has been characterized in the literature occurred in 2003 in the northwestern Mediterranean Sea with SSTs reaching 3–5 °C above the 1982–2016 reference period^4^. A MHW with similar intensity occurred off the coast of Western Australia in early 2011, persisting for >10 weeks^5,6^. The northeast Pacific Ocean experienced the largest MHW (often called “The Blob”) ever recorded between 2013 and 2015 with maximum SST anomalies of >6 °C off southern California^7^. Prominent MHWs also include the record high coastal ocean warming in the northwest Atlantic^8^, and the MHW in the Tasmanian Sea in 2015/16 that lasted >250 days^9^. MHWs also repeatedly occurred over the Western Pacific Warm Pool including the Great Barrier Reef in 1998, 2002, and 2016^10^.

The processes leading to the build-up, persistence and decay of a MHW are in general not well understood. Extreme ocean temperatures at the surface can be caused by large and small-scale oceanic forcing, atmospheric forcing or a combination of both, and the dominant mechanisms also depend on the location and season of occurrence. For example, the 2011 MHW off the coast of Western Australia was caused by predominant La Niña conditions in 2010/11 that drove the relatively warm Leeuwin Current southward along the west coast of Australia^5^. The northeast Pacific MHW from 2013 to 2015 was attributed to strong positive sea level pressure anomalies across the northeast Pacific that suppressed heat loss from the ocean to the atmosphere and caused anomalous and multi-year persistent high sea surface temperatures^12,13^.

## Impacts on physical, natural, and humans systems

So far the understanding of the impacts of MHWs on marine ecosystems has been opportunistically gained following a few recent events. Reported biological impacts range from geographical species shifts and widespread changes in species composition to harmful algal blooms, mass stranding’s of mammals and mass mortalities of particular species^14^. Two of the best studied MHWs with extensive ecological implications are the 2011 Western Australian MHW and the 2013–2015 northeast Pacific MHW. The 2011 Western Australian MHW resulted in an entire regime shift of the temperate reef ecosystem including a reduction in abundance of habitat-forming seaweeds, a subsequent shift in community structure and a southward distribution shift in tropical fish communities^6,15^. The warm blob in the northeast Pacific caused increased mortality of sea lions, whales and sea birds, very low ocean primary productivity, an increase in warm-water copepod species in the northern Californian region, and novel species compositions^16^. Another example is the MHW associated with the 2015/16 El Niño, which lead to the third mass coral bleaching event in recorded history with reported bleaching of over 90% of the surveyed reefs on the Great Barrier Reef in 2016^10^.

MHWs can also lead to significant political and socio-economic ramifications when affecting aquaculture or important fishery species. The MHW in the northwest Atlantic in 2012 has led to altered fishing practices and harvest patterns, price collapses of important fisheries and ultimately to intensified economic tensions between nations^8^. The warm blob in the northeast Pacific caused the closing of both commercial and recreational fisheries resulting in millions of dollars in losses among fishing industries^16^. MHWs can also perturb atmospheric conditions over land via teleconnections that may persist for multiple weeks to months. For example, anomalous high SSTs in the northeast Pacific have increased the probability for occurrence of the three consecutive dry winters in California during 2011–2014^12,17^. Therefore, MHWs may also have the potential to influence the predictability of extreme weather events over land.

## Important knowledge gaps

While long-term trends in global sea surface temperatures are relatively well known, an overview of past occurrences of MHWs and a mechanistic understanding of associated processes is currently missing. Notable exceptions are individual studies indicating that the extremely warm SSTs have become more common in more than one-third of the world’s coastal ocean^18^ over the past few decades and that the average interval between coral reef bleaching events globally has been halved over the last 25 years^19^. In addition, knowledge on the future progression of MHWs under different global warming scenarios and the risk for consecutive MHW events is currently lacking, despite strong evidence that global warming will increase the frequency and magnitude of land-based heat waves^2^.

**Fig. 2:**
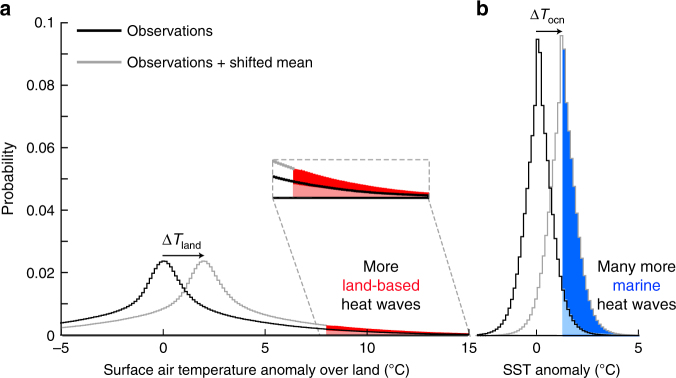
The effect of a simple shift towards a warmer climate on the probability of land-based and marine heat waves. **a** shows the observed distribution of the linearly detrended and deseasonalized local daily surface air temperature anomalies over land using the CRU-NCEP-v8 data set^21,22^; **b** as for **a** but for local daily sea surface temperature anomalies using NOAA’s daily Optimum Interpolation sea surface temperature data set^11^. Solid black lines show the distributions over the 1982–2016 period and solid gray lines indicate the same shape of the distributions, but the land is shifted by Δ*T*_land = _2°C and the ocean by Δ*T*_ocn_ = 1.33 °C. Here we assume Δ*T*_land_/Δ*T*_ocn = _1.5^20^. A heat wave is defined as temperature exceeds the 95th percentile (red and blue shaded areas). The inset highlights the changes in land-based heat waves

A thought experiment using observed local daily land and sea surface temperature distributions might provide some guidance about the progression of MHWs under global warming (Fig. 2). A simple shift towards a warmer climate of the entire land and sea surface temperature distribution causes an increase in the probability of occurrence of both land-based and marine heat waves. Climate models consistently show that warming is about 1.5 times larger over land (Δ*T*_land_ in Fig. 2) than over the ocean (Δ*T*_ocn_), independent of the level of global warming^20^. Intuitively, this points towards a larger increase in the probability of occurrence of heat waves over land than over the ocean. However, due to the narrow sea surface temperature distribution, the smaller temperature increase at the ocean surface still leads to a disproportionally larger increase in the probability of MHWs than of land-based heat waves. Clearly, changes in the higher moments of the probability temperature distribution like increasing/decreasing variance or skewness can also produce changes in the likelihood of extreme events without changes in the mean, and could modify the conceptual picture drawn in Fig. 2.

Major scientific progress in probabilistic event attribution research makes it now possible to attribute the risk of individual extreme climate events over land to human-induced climate change and past carbon emissions^23^. However, so far only a couple of studies applied an attribution framework to MHWs^9,24^. These studies conclude that the severity of all but one MHW was increased due to climate change. One study even concluded that the occurrence of the unusually warm water off the coast of Alaska in 2016 would not have even been possible without climate change. Further research into this attribution field is needed to assign losses and damages associated with MHWs to country-level responsibility^25^.

The vulnerability of marine ecosystems to MHWs depends not only on the duration and magnitude of the extreme event, but also on the sensitivity and adaptive capacity of the ecosystem to these events. Risk assessments for marine ecosystems have focused largely on mean changes in temperature. Notable exceptions are the assessment of risks for coral reef systems. As a result, there is currently limited ability to project the effects of MHWs on the future of marine ecosystems and to advise society on effective adaption strategies.

Observations and model simulations also demonstrate that other factors such as ocean acidification and deoxygenation are putting stress on marine ecosystems. However, little is known about possible ocean “hotspots”, where extreme events in temperature, acidification and oxygen occur at the same time or successively (i.e., compound events).

## The way forward

A number of recent developments may foster significant progress over the next few years. First, novel physical and biogeochemical data synthesis (e.g., remote sensing products, surface ocean CO_2_ Atlas, profiling floats equipped with biogeochemical sensors) have become available over the last few years promising a breakthrough in assessing heat waves and extreme events in other potential ocean stressors. Second, the horizontal and vertical resolution of the Earth System Models, especially of the ocean components, and the computational power to run simulations at high resolution has continuously increased, so that it is now feasible to investigate MHWs in sufficient high resolution at the global scale. Third, the level of sophistication of marine ecosystem-biogeochemical models in these global Earth System models has improved to a stage permitting us to investigate and simulate changes in MHWs and extreme events in other individual and combined potential ocean ecosystem stressors. Fourth, major progress in probabilistic event attribution research over the last few years allow us to tackle the question whether and to what extent external climate drivers such as human-induced climate change alter the likelihood of ocean extreme events. And finally, recent global compilations of ecosystem relevant parameters, as well as concepts and empirical models of the functioning of organisms and their responses to environmental changes will give the opportunity to assess and quantify risks of ocean extreme events for marine ecosystems in novel ways. However, retrospectives studies, innovative laboratory experiments and interdisciplinary approaches are needed to ultimately understand the impact of MHWs on individual organisms, ecosystems, and their socioeconomic services.
